# Echocardiographic measurements of left ventricular dimensions and function in newborns with omphalocele and pulmonary

**DOI:** 10.1186/s12887-023-04418-y

**Published:** 2023-11-22

**Authors:** Si-Si Yang, Wen-Chang Huang, Peng Wang, Fang-Qi Gong, Tai-Xiang Liu, Jin-Fa Tou, Deng-Ming Lai

**Affiliations:** 1 Department of Neonatal Surgery, Children’s Hospital, Zhejiang University School of Medicine, National Clinical Research Center for Child Health, 3333 Binsheng Rd, Hangzhou, Zhejiang China; 2 Department of Cardiology, Children’s Hospital, Zhejiang University School of Medicine, National Clinical Research Center for Child Health, 3333 Binsheng Rd, Hangzhou, Zhejiang China; 3 Department of NICU, Children’s Hospital, Zhejiang University School of Medicine, National Clinical Research Center for Child Health, 3333 Binsheng Rd, Hangzhou, Zhejiang China

**Keywords:** Omphalocele, Pulmonary hypertension, Echocardiogram, Left ventricle, Prognosis

## Abstract

**Purpose:**

The purpose of this study was to explore echocardiographic parameters of the left ventricle (LV) in relation to the outcomes of omphalocele neonates with pulmonary hypertension (PH).

**Methods:**

This retrospective study was conducted among omphalocele patients with PH born from 2019 to 2020. Patients in this study did not have additional severe malformations or chromosomal aberrations. Patients who died under palliative care were excluded. The echocardiographic parameters of LV were obtained within 24 h after birth. Clinical and outcomes data were recorded, echocardiograms evaluated for left ventricular internal dimension in end-diastole (LVIDd), end-diastolic volume (EDV), stroke volume (SV) and cardiac output index (CI), among others.

**Results:**

There were 18 omphalocele newborns with PH, of whom 14 survived and 4 died. Both groups were comparable in the baseline characteristics. Non-survival was associated with a smaller LV [LVIDd (12.2 mm versus15.7 mm, *p* < 0.05), EDV (3.5 ml versus 6.8 ml, *p* < 0.05)] and with worse systolic function [SV (2.3 ml versus 4.2 ml, *p* < 0.05), and CI (1.7 L/min/m^2^ versus 2.9 L/min/m^2^, *p* < 0.01)].

**Conclusion:**

In the cohort of omphalocele patients with PH, lower LVIDd, EDV, SV and CI were associated with mortality.

**Level of evidence:**

Level III.

## Introduction

Pulmonary hypertension (PH) has been known to adversely affect outcome in neonates diagnosed with omphalocele [1, 2]. This is intimately associated with pulmonary vascular hypoplasia (delayed or impaired relaxation) [3], leading to respiratory distress and labile hypoxemia [4, 5]. Through prompt diagnosis and appropriate treatment with inhaled vasodilators like sildenafil and nitric oxide, the outcomes of some patients were improved, but others were not.

Frequently, echocardiographic assessment focus on the right ventricle (RV) primarily affected by PH with subsequent involvement of the left ventricle (LV) due to ventricular interdependance [6]. However, there is limited information about the left ventricular function and its effect on outcomes in omphalocele neonates with PH.

This study examined echocardiographic parameters associated with the prognosis of omphalocele with PH and found indices related to LV to be of interest. Through study of this cohort, we aimed to analyze the echocardiographic parameters of LV in the early stage after birth and predict the prognosis in patients of omphalocele with PH.

## Methods

### Study population

With the approval of the Institutional Review Board of the Children’s Hospital of Zhejiang University school of medicine (approval number of 2021-IRB-012), the data of all omphalocele neonates who diagnosed with PH treated at our center from January 2019 to December 2020 were retrospectively analyzed. The early echocardiogram was important in postnatally evaluation, but PH was diagnosed based upon echo on or after day 2 of life, with the echocardiogram-estimated pulmonary artery pressure (PAP) > 2/3 systemic blood pressure and/or right-to-left or bidirectional flow at ductal and/or atrial level [6, 7]. Echocardiography was performed to assess PAP (PAP = 4 × (tricuspid regurgitation velocity max)2 + right atrium pressure), which reflects the right ventricular systolic blood pressure when there is no right ventricle outflow tract obstruction [8]. The echocardiographic parameters were obtained by the Philips iE33 (Philips Ultrasound, Bothell, WA, USA). Giant omphalocele (GO) was defined as a fascial defect in abdominal wall, with > 75% herniated liver in the sac [1]. A total of 20 omphalocele patients with PH were born during this period. Patients who died due to the management of palliative care were excluded. In total, 14 surviving infants with PH and 4 dead infants with PH were compared in this study. All the 18 patients presented with signs of clinical deteriorations such as increased respiratory need, and were treated with repeated echocardiograms and pulmonary vasodilator therapy.

### Date collection

This was a retrospective study of medical records and echocardiogram images. The outcome measure was survival rate at 1st hospitalization. Medical records were reviewed to obtain patient demographics, including gender, gestational age, birth weight, length, head circumference, chest circumference, Apgar score at 1 and 5 min, whether ventilation support was required at birth, content of the sac, cardiovascular management, and maternal Information. All postnatal echocardiograms were performed by a certified echocardiographic technician within 24 h of birth. And obtained echocardiographic parameters of LV included interventricular septum in end-systole (IVSs), and in end-diastole (IVSd), left ventricular internal dimension in end-diastole (LVIDd), and in end-systole (LVIDs), left ventricular posterior wall in end-systole (LVPWs) and in end-diastole (LVPWd), end-diastolic volume (EDV), end-systolic volume (ESV), ejection fraction (EF), fractional shortening (FS), stroke volume (SV), and cardiac index (CI).

### Data analysis

Categorical variables were presented in frequency and percentage terms. The Fisher’s exact test was used to compare categorical data. Continuous variables were presented as median and interquartile ranges. The Mann–Whitney U test was used to analyze continuous data. Statistical significance was taken as values of two-sided *P* < 0.05. Statistical analyses were performed using SPSS software version 26.0.

## Results

There were 20 omphalocele neonates with PH diagnosed and treated at our institution during the study period between January 2019 and December 2020. Two patients were excluded from the analyses due to managed by palliative care, and died at 24 days and 25 days, respectively. The remaining 18 patients all had available echocardiograms, which could be used to diagnose PH. None of them had severe congenital malformations or abnormal chromosomes. In all, 15 (75.0%) neonates were GO and 13 (65.0%) underwent staged closure. 3 (16.7%) patients required nitric oxide therapy, 17 (85.0%) and 7 (38.9%) patients were treated with pulmonary vasodilator therapy with sildenafil or bosentan, and 14 (77.8%) survived to discharge.

Baseline demographic characteristics of entire patient cohort stratified according to outcomes at 1st hospitalization are shown in Table 1. Many patients were born with normal conditions, especially those in the survival group. Birth chest circumference and requirement of intubation at birth were considered with respiratory system failure [9–12], but two-sided P-vales were 0.097 and 0.108 (> 0.05), respectively. And there were no significant differences in patient or maternal characteristics between infants who survived to discharge and those who did not. These patients were diagonsed with PH at the age of 2–26 days and treated with pulmonary vasodilator at the age of 2–30 days, including nitric oxide, sildenafil and bosentan.

**Table 1:** Baseline characteristics in the survival and death groups of patients with PH.

	Survived (n = 14)	Did not survive (n = 4)	*P*-value
Patient information			
Gender (male)	8 (57.1%)	2 (50.0%)	1.000
GA (weeks), median (IQR)	38.0 (37.5–38.9)	36.3 (32.4–38.7)	0.420
Preterm (< 37 weeks GA)	3 (21.4%)	2 (50.0%)	0.533
BW (kg), median (IQR)	3.0 (2.5–3.4)	2.4 (1.5–2.9)	0.089
Low BW (< 1500 g)	3 (21.4%)	2 (50.0%)	0.533
BL (cm), median (IQR)	48.5 (46.8–50.0)	44.0 (40.8–48.8)	0.146
BH (cm), median (IQR)	34.0 (32.5–35.0)	32.5 (28.4–34.0)	0.186
BC (cm), median (IQR)	32.0 (30.0-33.3)	28.5 (25.5–31.5)	0.097
1-min Apgar	8.0 (7.0–10.0)	7.5 (5.3–9.8)	0.440
Low Apgar score (< 7)	2 (14.3%)	2 (50.0%)	0.197
5-min Apgar	10.0 (9.8–10.0)	9.5 (8.25-10.0)	0.311
Intubation at birth	1 (7.1%)	2 (50.0%)	0.108
GO	11 (78.6%)	4 (100%)	-
Content of omphalocele			
Liver	11 (78.6%)	4 (100%)	-
Stomach	5 (35.7%)	3 (75.0%)	0.275
Spleen	3 (21.4%)	2 (50.0%)	0.533
Stage closure	9 (64.3%)	4 (100%)	-
Maternal Information			
Maternal age (years)	30.0 (27.5–36.3)	29.5 (26.5–31.8)	0.749
Adverse pregnancy	1.0 (0–2.0)	1.5 (1.0-2.8)	0.379
Pregnancy-induced hypertension	1 (7.1%)	1 (25.0%)	0.405
Gestational diabetes	2 (14.3%)	2 (50.0%)	0.197
Medication use in pregnancy	9 (64.3%)	2 (50.0%)	1.000
PH			
Age at diagnosis (days)	3.5 (2.8–9.5)	2.5 (2.0-4.5)	0.232
Pulmonary vasodilator therapy			
Age at therapy (days)	7.5 (4.8–19.0)	5.5 (3.3–9.3)	0.394
Nitric oxide	2 (14.3%)	1 (25%)	1.000
Sildenafil	13 (92.9%)	4 (100%)	-
Bosentan	7 (50%)	0	-

GA: gestational age; BW: birth weight; BL: birth length; BH: birth head circumference; BC: birth chest circumference; GO: giant omphalocele; IQR: interquartile range; PH: pulmonary hypertension

**Fig. 1:**
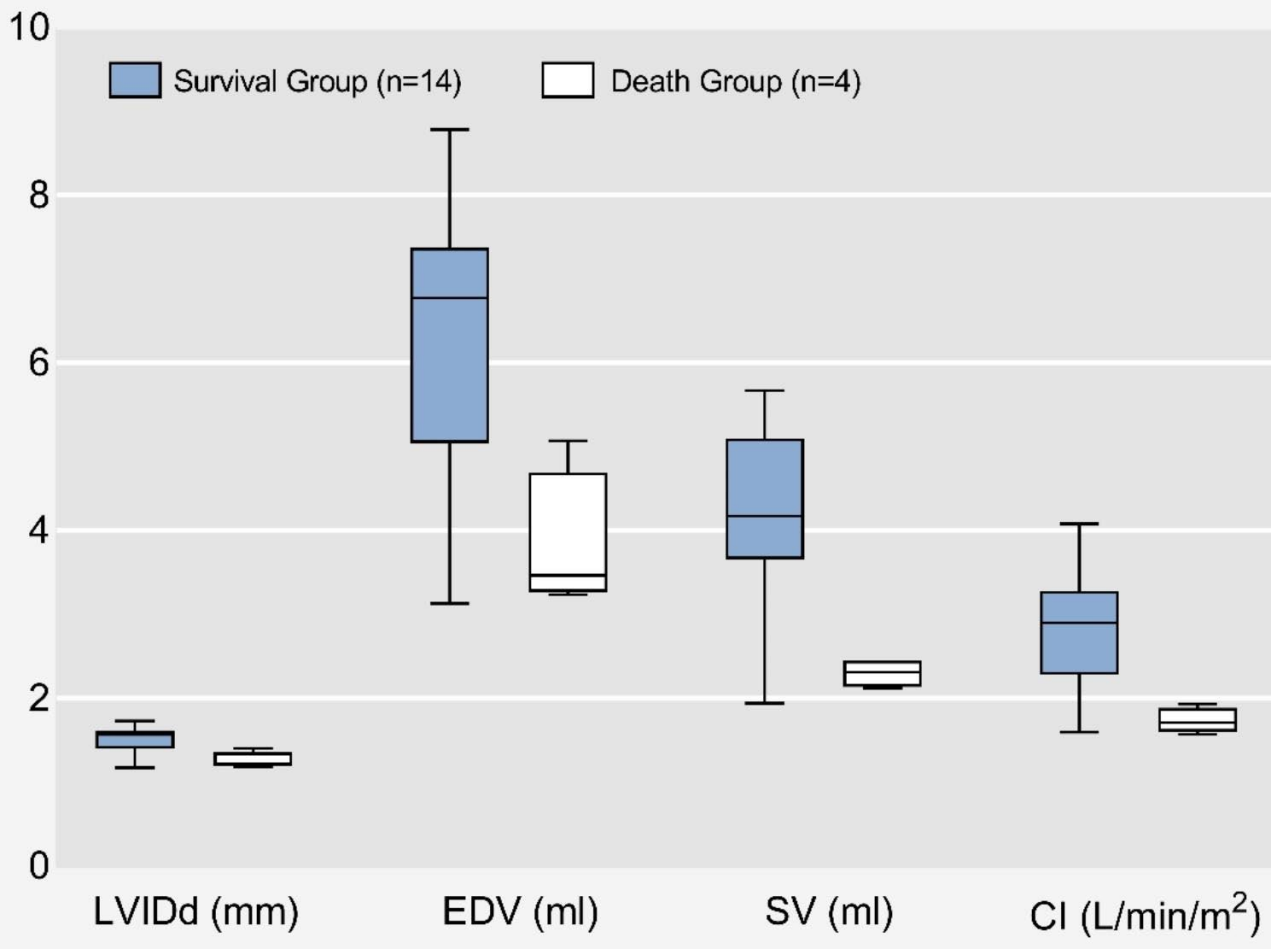
A box plot showing echocardiographic parameters (LVIDd, EDV, SV, CI) in groups of omphalocele with PH who died and survived

Table 2 compares echocardiographic parameters of the LV within the first 24 h after birth in survival and death groups of patients with PH. There were significant differences between the two groups with respect to LVIDd (15.7 mm versus 12.2 mm, *p* < 0.05), EDV (6.8 ml versus 3.5 ml, *p* < 0.05), SV (4.2 ml versus 2.3 ml, *p* < 0.05), and CI (2.9 L/min/m^2^ versus 1.7 L/min/m^2^, *p* < 0.01). There were no observed association between IVSs, IVSd, LVIDs, LVPWs, LVPWd, ESV, EF, and FS in two groups. Figure 1 depicts a box plot of LVIDd, EDV, SV and CI in the two groups of patients: survival patients with PH and death patients with PH.

**Table 2:** Echocardiographic parameters within 24 h after birth in the survival and death groups of patients with PH.

	Survived(n = 14)	Did not survive(n = 4)	*P*-value
IVSs (mm), median (IQR)	4.7(3.8–5.6)	4.1 (2.6–4.7)	0.242
IVSd (mm), median (IQR)	4.1(3.5–4.4)	3.3 (2.6–3.6)	0.088
LVIDs (mm), median (IQR)	10.0(8.9–11.2)	8.5 (7.3–10.4)	0.150
LVIDd (mm), median (IQR)	15.7(14.0-16.2)	12.2 (11.9–13.6)	**0.029**
LVPWs (mm), median (IQR)	3.8(3.1–4.6)	3.4 (2.9-4.0)	0.366
LVPWd (mm), median (IQR)	2.9(2.7–3.7)	3.0 (2.2–3.3)	0.286
ESV (ml), median (IQR)	2.1(1.5–2.8)	1.3 (0.9–2.3)	0.151
EDV (ml), median (IQR)	6.8(5.0-7.4)	3.5 (3.3–4.7)	**0.029**
SV (ml), median (IQR)	4.2(3.7–5.1)	2.3 (2.1–2.4)	**0.011**
EF (%), median (IQR)	64.7(60.0-73.9)	62.2 (51.0-73.1)	0.457
FS (%), median (IQR)	32.5(29.4–39.6)	30.2 (23.6–38.5)	0.396
CI (L/min/m^2^), median (IQR)	2.9(2.3–3.3)	1.7 (1.6–1.9)	**0.008**

IVSs, interventricular septum in end-systole; IVSd, interventricular septum in end-diastole; LVIDs, left ventricular internal dimension in end-systole; LVIDd, left ventricular internal dimension in end-diastole; LVPWs, left ventricular posterior wall in end-systole; LVPWd, left ventricular posterior wall in end-diastole; ESV, end-systolic volume; EDV, end-diastolic volume; SV, stroke volume; FS, fractional shortening; CI, cardiac index; IQR, interquartile range

Bold values indicate two-sided *P*-values < 0.05

## Discussion

In this study, we analyzed a cohort of neonates with omphalocele who had echocardiographic evidence of PH, and investigated the risk factors for death. They all underwent standardized care at a single center. All patients with PH were treated with pulmonary vasodilator therapy (sildenafil or bosentan), and some of these patients were treated with nitric oxide therapy (3/18, or 16.7%). The outcome at 1st hospitalization was not significantly associated with patient or maternal characteristics, such as birth weight, gender, gestational age, or maternal age, adverse pregnancy. The results of this single-center retrospective study demonstrated that those neonates who died have worse echocardiographic parameters of LV within 24 h of birth, as reflected by mean lower LVIDd, EDV, SV, and CI, compared with survivors. CI in survival group was 2.9(2.3–3.3), that is, if the ratio of the cardiac output to the body surface area was close to 3.0. The patients who did not survive had a significantly impaired CI with a median value < 2.0. Parameters of LV at initial echocardiography were significantly associated with mortality in the omphalocele with PH population.

The survival rates of newborns with omphalocele have been on the rise over the past few decades due to the improvements of parenteral nutrition, surgery and intensive care [13]. However, omphalocele remains a cause for concern, born with varying degrees of congenital malformation, and neonatal survival with severe PH remains unsatisfactory [14, 15]. Immediately, increased pulmonary pressures after birth in newborns with omphalocele has been observed in many case reports [16], which highlights the importance of early and routine echocardiography [2]. Many previous studies have described pulmonary hypoplasia through the measurements of lung volume in this patient population [17–19]. While more research is needed to investigate the mechanisms of PH and pulmonary hypoplasia, the disturbed transition to extrauterine life may be related to increased pulmonary pressure [20]. Multiple mechanisms are at work at birth to reduce PAP and ensure a smooth transition of pulmonary vascular in newborns. Starting with the stimulation of pulmonary ventilation [21], accompanied by the closure of foramen ovale and ductus arteriosus, the rapid structural remodeling of the entire pulmonary bed finally completes the the final phase of pulmonary vascular transition [22].

While previous studies focused on pulmonary artery size and pressure [6], our aim was to assess the specific contribution of the initial poor LV echocardiographic parameters to mortality. This study suggests that worse echocardiographic parameters of LV within 24 h of birth is associated with the increased mortality in omphalocele patients with PH, which may be related to LV hypoplasia. There are some pathogenic mechanisms that may describe the occurrence of LV hypoplasia in omphalocele patients with severe PH. One hypothesis is that thoracic collapse and increased pressure in RV cause a continuous mechanical compression of the left heart, preventing full growth of LV [23]. Another hypothesis is that persistent mechanical compression and reduced pulmonary vasculature led to the reduction of pulmonary blood flow, resulting in decreased preload of the LV, which in turn leads to left ventricular hypoplasia [24, 25].

If the mechanical compression leads to LV hypoplasia, early reduction of viscera may elevate the diaphragm and worsen LV disorder, making the condition more difficult in patient with omphalocele. Surgery closure must be delayed secondary to cardiac conditions and PH, which is conducive to physiologic optimization. Furthermore, left ventricular hypoplasia may be associated with refractory treatment of PH. Therapies included sildenafil, inhaled nitric oxide, and bosentan have been successfully used to treat PH in some patients [26–28], but others not. In this group of omphalocele patients, outcomes were not improved by pulmonary vasodilator in 4 patients. PH, RV failure combined with LV hypoplasia may result in severe conditions that does not respond to conventional treatment. Several strategies of gentle ventilation, reducing oxygen exposure, inodilators, and PGE1 may enhance LV function and increase oxygenation, improving outcome in omphalocele with PH. Many reports have advocated the management of pulmonary vasodilators, high-frequency ventilation, and extracorporeal membrane oxygenation in omphalocele newborns with PH to eliminate RV dysfunction [1, 2, 29]. Addressing RV dysfunction is always the focus of attention, but there is not currently a treatment strategy for LV disorder. The importance of global biventricular function should be emphasized when tailoring therapies for high-risk newborns, and which treatment options are more effective needs to be addressed in future studies.

In addition, there was a significant association between liver-containing defects and the development of PH [27, 30–32], as well as between the need for intubation at birth and PH [2, 27]. However, we did not observe significant differences between the content of sac and increased mortality before discharge, or between the need for intubation at birth and mortality.

All clinical data came from a single institution and the management were consistent during the relatively limited time. Our study was restricted by the inherent limitations of retrospective research and the small sample size. Encouragingly, initial poor echocardiographic parameters of the LV, measured by non-invasive and widely used echocardiography, was associated with prognosis.

## Conclusions

Echocardiographic measurements provide reliable parameters for forecasting poor prognosis of omphalocele patients with PH. Particularly, mean lower LVIDd, EDV, SV, and CI were associated with mortality in omphalocele patients with PH. Further studies are wanted to improve patient outcomes, and more precise echocardiographic assessments for forecasting outcomes are also necessary to tailor the treatments of omphalocele patients with serious PH.

## Data Availability

Data analysed or created in this study is involved in this publish article.

## Abbreviations

PH: Pulmonary hypertension
RV: Right ventricle
LV: Left ventricle
PAP: Pulmonary artery pressure
GO: Giant omphalocele
IVSs: Interventricular septum in end-systole
IVSd: Interventricular septum in end-diastole
LVIDd: Left ventricular internal dimension in end-diastole
LVIDs: Left ventricular internal dimension in end-systole
LVPWs: Left ventricular posterior wall in end-systole
LVPWd: Left ventricular posterior wall in end-diastol
EDV: End-diastolic volume
ESV: End-systolic volume
EF: Ejection fraction
FS: Fractional shortening
SV: Stroke volume
CI: Cardiac index
